# Regular physical exercise training assists in preventing type 2 diabetes development: focus on its antioxidant and anti-inflammatory properties

**DOI:** 10.1186/1475-2840-10-12

**Published:** 2011-01-28

**Authors:** Edite Teixeira-Lemos, Sara Nunes, Frederico Teixeira, Flávio Reis

**Affiliations:** 1 Unit of Therapeutics, Laboratory of Pharmacology and Experimental Therapeutics, IBILI, Medicine Faculty, University of Coimbra, Portugal

## Abstract

Diabetes mellitus has emerged as one of the main alarms to human health in the 21st century. Pronounced changes in the human environment, behavior and lifestyle have accompanied globalization, which resulted in escalating rates of both obesity and diabetes, already described as diabesity. This pandemic causes deterioration of life quality with high socio-economic costs, particularly due to premature morbidity and mortality. To avoid late complications of type 2 diabetes and related costs, primary prevention and early treatment are therefore necessary. In this context, effective non-pharmacological measures, such as regular physical activity, are imperative to avoid complications, as well as polymedication, which is associated with serious side-effects and drug-to-drug interactions.

Our previous work showed, in an animal model of obese type 2 diabetes, the Zucker Diabetic Fatty (ZDF) rat, that regular and moderate intensity physical exercise (training) is able, per se, to attenuate insulin resistance and control glycaemia, dyslipidaemia and blood pressure, thus reducing cardiovascular risk, by interfering with the pathophysiological mechanisms at different levels, including oxidative stress and low-grade inflammation, which are key features of diabesity.

This paper briefly reviews the wide pathophysiological pathways associated with Type 2 diabetes and then discusses in detail the benefits of training therapy on glycaemic control and on cardiovascular risk profile in Type 2 diabetes, focusing particularly on antioxidant and anti-inflammatory properties. Based on the current knowledge, including our own findings using an animal model, it is concluded that regular and moderate intensity physical exercise (training), due to its pleiotropic effects, could replace, or at least reduce, the use of anti-diabetic drugs, as well as of other drugs given for the control of cardiovascular risk factors in obese type 2 diabetic patients, working as a physiological "polypill".

## Introduction

Type 2 diabetes mellitus (T2DM) achieved proportions of a real epidemic and, according to the International Diabetes Federation (IDF), the disease now affects 246 million people worldwide and is expected to affect about 380 million by 2025 [1]. This panorama is even more dramatic if considered that over the past 20 years its prevalence has increased dramatically among children and adolescents. As such, the incidence/prevalence of serious diabetic complications (which includes cardiovascular disease, kidney failure, blindness and amputations) as well as the premature death, will unequivocally deteriorate life quality and exacerbate health costs, unless more effective primary and secondary pharmacological and non-pharmacological (lifestyle interventional) strategies become more widely available and implemented. The therapeutic arsenal now available to manage T2DM has proved inefficacy to prevent the rise in incidence of cardiovascular events, the leading cause of morbidity and early mortality of diabetic patients. The improvement of cardiovascular profile will depend not only on the reduction of glycosylated hemoglobin (HbA1c) levels, but also of other factors, such as blood pressure. The Steno-2 study [2] clearly demonstrated that only intensive multifactorial intervention, involving pharmacological measures and lifestyle modifications, could promotes sustained beneficial effects on rates of death and cardiovascular disorders in T2DM patients.

Several prospective studies have associated time spent in sedentary activities, such as TV watching and computer or video-games use, with increasing obesity [3] and T2DM development [4]. Thus, the low level of physical activity (sedentarism) has been considered a risk factor for early mortality, in parallel with smoking habits, arterial hypertension and dyslipidaemia. The regular practice of moderate intensity physical exercise (training) showed capacity to reduce body weight, improve insulin sensitivity, increase circulating levels of high-density lipoprotein (HDL), decrease triglyceride levels and normalize blood pressure [5,6].

This paper will review the effects of regular practice of an aerobic exercise program of moderate intensity (training) in the prevention of T2DM or attenuation of its progression, based on the present literature as well as on our knowledge using an experimental model of obese T2DM. We will firstly focus on the effects at different risk factors related to insulin resistance (IR) and glucose intolerance stages, which precedes the onset of sustained T2DM; thereafter, the review will focused on the effects promoted by exercise training on oxidative stress and low-grade inflammation, which play a key role on the onset and progression of diabetes.

## Natural history of T2DM: role of oxidative stress and inflammation

T2DM is a complex heterogeneous group of metabolic conditions characterized by increased levels of blood glucose due to impaired insulin action and/or secretion [7]. Physiologically, the pancreatic β-cells constantly synthesize insulin, regardless of blood glucose levels. Insulin is stored within vacuoles and released once triggered by an elevation of the blood glucose level. Insulin is the key hormone concerning the regulation of glucose uptake from blood into most cells, including skeletal muscle cells and adipocytes. Insulin is also the major signal for conversion of glucose to glycogen for internal storage in liver and skeletal muscle cells. A drop in the blood glucose level results in decrease of insulin release from β-cells and in increase of glucagon release from α-cells, which stimulates glycogen to glucose conversion. Following an overnight fast, glucose is largely produced by glycogenolysis and gluconeogenesis.

There are three key defects in the onset of hyperglycemia in T2DM: increased hepatic glucose production, diminished insulin secretion and impaired insulin action [8]. Insulin resistance refers to a suppressed or delayed response to insulin and is generally a post-receptor phenomenon, due to a defect in cells that respond to insulin, rather than on insulin production.

Insulin resistance in muscle and liver, together with β-cell failure, are pivotal pathophysiologic defects in T2DM. It is now recognized that β-cell failure occurs much earlier and is more severe than previously thought. Subjects in the upper tertile of impaired glucose tolerance (IGT) are maximally or near-maximally insulin resistant and have lost over 80% of their β-cell function. In addition to muscle, liver and β-cells, the fat cell (accelerated lipolysis), the gastrointestinal tract (incretin deficiency/resistance), the α-cell (hyperglucagonemia), the kidney (increased glucose reabsorption) and the brain (insulin resistance) all play important roles in the development of glucose intolerance in type 2 diabetic individuals.

The insulin resistance observed in obese T2DM patients is secondary to changes in insulin receptors signal transduction, arising from genetic and/or environmental factors, such as excessive caloric consumption, sedentary lifestyle and obesity [9]. T2DM emerges when these change are associated with a progressive decrease in the secretory capacity of pancreatic beta cells (relative insulinopenia). It is a silent disease, in which the loss of secretory capacity begins years before the clinical diagnosis. In the phase of insulin resistance, glucose intolerance or glycaemia rise may occur in the unfed state. As long as the β-cells are able to augment insulin secretion to compensate insulin resistance, glucose tolerance remains normal. However, with time, the β-cells begin to fail and the postprandial plasma glucose levels (initially) and the fasting plasma glucose concentration (subsequently) begin to rise, leading to the onset of overt diabetes [9,10].

It has been suggested that chronic hyperglycaemia or even the intermittent blood glucose elevations observed in patients with apparent acceptable glycaemic control may contribute to the development of macro and/or microvascular complications [11,12]. However, many different pathophysiological pathways may be simultaneously activated, which includes oxidative stress and low-grade inflammation.

### Role of oxidative stress in the progression of T2DM

It has been shown that reactive oxygen species (ROS) are produced in various tissues under diabetic conditions, by the several mechanisms, such as non-enzymatic glycosylation reactions, electron transport chain in the mitochondria and membrane-bound nicotinamide adenine dinucleotide phosphate (NADPH) oxidase [13]. Several lines of evidence support a close association between oxidative stress and diabetes evolution, revealing that oxidative stress secondary to hyperglycaemia and hyperlipidaemia occurs before the appearance of clinical manifestations of late diabetes complications, suggesting a key role in the pathogenesis of the disease. Therefore, insulin resistance and pancreatic β-cell dysfunction, which are hallmarks of type 2 diabetes, are modulated by ROS [14-16]. Under diabetic condition, chronic hyperglycaemia may induce large amounts of ROS that are responsible for the progressive dysfunction of β cells, worsening insulin resistance and further promoting relative insulin deficiency ROS [17]. It was previously reported that ROS disrupt insulin-induced cellular redistribution of insulin receptor substrate-1 (IRS-1) and phosphatidylinositol 3-kinase (PI3K), thus impairing insulin-induced glucose transporter type 4 (GLUT4) translocation in 3T3-L1 adipocytes [14]. The increase of intracellular free fatty acids (FFA), in turn, leads to a decreased translocation of the glucose transporter subtype 4 (GLUT4) to the plasma membrane, leading to insulin resistance in muscle and adipose tissue [18]. In this context, insulin resistance may be considered a compensatory mechanism that protects the cells against further insulin-stimulated glucose and fatty acid uptake and, therefore, oxidative damage. Many studies have previously suggested that ß-cell dysfunction results from prolonged exposure to high glucose and FFA levels, or a combination of both [14,15]. Beta cells, in particular, are particularly sensitive to ROS because they are low in free-radical quenching (antioxidant) enzymes such as catalase, glutathione peroxidase, and superoxide dismutase [19,20]. The ROS formed will also indirectly damage cells by activating a variety of stress-sensitive intracellular signaling pathways, including Nuclear factor-kB (NF-kB), mitogen-activated protein kinase p38 (p38MAPK), kinases JunNH2- terminal/kinases of proteins activated by stress (JNK/SAPK), hexosamines, protein kinase C (PKC) and polyol pathway [15,21,22]. The activation of these cellular signaling cascades is linked not only with the development of diabetic complications but also with the insulin resistance and pancreatic β cell dysfunction. Among the signaling cascades, the NF-kB pathway plays a central role as intermediary of immune and inflammatory responses. This nuclear factor is responsible for regulating the expression of a large number of genes, including those related to diabetes complications, such as the vascular endothelial growth factor (VEGF) [20]. Being an intracellular signaling pathway target of hyperglycaemia and ROS, its activation may also be induced by endogenous and exogenous stimuli, in addition to those above mentioned, such as excess of FFA, tumour necrosis factor α (TNF-α), interleukin 1β (IL-1β) and other pro-inflammatory cytokines, advanced glycation endproducts (AGE) related to receptor for AGE (RAGE), p38MAPK, DNA damage, viral infection and ultraviolet radiation [21].

### Role of inflammation in the progression of T2DM

Obesity, as a result of inactivity in combination with overeating, plays a key role in the development of pancreatic beta-cell dysfunction and in insulin resistance. An increased mass of stored triglycerides (TGs), especially in visceral or deep subcutaneous adipose stores, leads to large adipocytes, that are resistant to insulin-evoked lipolysis suppression, resulting in increased release of FFA and glycerol. This "dyslipidaemic phenotype of diabetes", characterized by increased content of TGs and oxidized low density lipoproteins (ox-LDL), together with decreased levels of HDL, is responsible for the lipotoxicity profile of diabetes. Lipotoxicity has been used to describe the deleterious effect of tissue fat accumulation on glucose metabolism and includes the notion that increased plasma FFA/intramyocellular levels of toxic lipid metabolites (such as long-chain fatty acyl CoAs, diacylglycerol and ceramides) play a role in the pathogenesis of muscle/liver insulin resistance.

In addition, fat cells produce adipocytokines, which go through distant sites (such as muscle, liver and arterial tissue), where exert deleterious effects on metabolism and vascular function. Adipose tissue of obese and type 2 diabetic individuals is infiltrated by mononuclear cells and is in a state of chronic inflammation [23]. The adipocytes and infiltrated macrophages secrete pro-inflammatory/pro-thrombotic cytokines, such as the TNF-α, interleukin-6 (IL-6), resistin, adipsin, acylation-stimulating protein (ASP), plasminogen activator inhibitor 1 (PAI-1) and angiotensinogen, that promote atherogenesis and cause insulin resistance. Adipocytes also produce adiponectin, a potent insulin-sensitizing and anti-atherogenic cytokine, now included in a vast group of substances named adipokines or adipocytokines. Low adiponectin levels have been correlated with visceral obesity and whole-body insulin sensitivity [24]. This fat cell hormone acts as an insulin sensitizer, inhibiting TGs formation in liver and stimulating fatty acid oxidation in muscle in an 5' adenosine monophosphate-activated protein kinase (AMPK) and peroxisome proliferators activated receptor alpha (PPAR-α)-dependent manner [25]. Despite their apparent importance in the insulin resistance syndrome, aforementioned adipokines are just examples of a family of adipocyte-derived factors that modulate insulin resistance and systemic inflammation. Besides new adipokines, also certain myokines now appear to affect insulin sensitivity and inflammatory responses. As such, the list of insulin (de)sensitizing proteins and cytokines is still far from complete. The secretion of citokines depends not only on the amount of adipose tissue but also of its location, being visceral or intra-abdominal fat more harmful than subcutaneous fat. The pro-inflammatory effects of cytokines are felt at the intracellular levels of signaling cascades and involves the pathways of NF-κB and JNKs [26,27].

The increase of pro-inflammatory cytokines, associated with the dyslipidaemic profile in T2DM, may not only modulate the function of pancreatic beta cells but also their survival. Several studies showed that exposure of β cells to high levels of saturated fatty acids and lipoproteins undertake to their death, and this effect is accelerated by hyperglycaemia, demonstrating that lipotoxicity and glucotoxicity, in concert, determinate beta-cell failure [28-31].

The briefly preceding review of the key pathophysiological mechanisms of T2DM highlights several relevant aspects for the therapeutics. First, effective treatment of T2DM will require combination of multiple drugs to correct the various pathophysiological defects. Second, treatment should not be simply directed to HbA1c reduction, but also based upon known pathogenic abnormalities, which includes the preservation of antioxidant and anti-inflammatory capacity. Third, since progressive β-cell failure is to be prevented, therapy must be started early in the natural history of type 2 diabetes.

## Exercise training as a therapeutic modality in T2DM

Physical inactivity has been identified as a stronger predictor of chronic diseases even when compared with traditional risk factors, such as hypertension, hyperlipidaemia, diabetes and obesity. Moreover, regular physical activity appears to protect against premature death, independently of obesity.

Several studies, clinical and experimental, have been assessing the role of regular physical activity (training) on cardiovascular and cardiometabolic disorders, including on diabetes. Although results from studies using animals cannot be directly extrapolated for humans, animal models of T2DM could offer excellent opportunities to evaluate experimental conditions and to assess tissues that cannot be tested in humans, thus improving the knowledge about the endocrine, metabolic and morphological changes underlying the pathogenic mechanisms of the disease and the treatment options.

In the following topics we will review the benefits of a particular physical exercise (training) in the wide pathophysiological aspects associated with T2DM, focusing on antioxidant and anti-inflammatory properties, based on the information already available in the literature, from both clinical and experimental studies, and in particular on the data obtained from our own experiments using an animal model of obese T2DM, the Zucker Diabetic Fatty (ZDF *fa/fa*) rats.

In order to not repeat the information alongside the text, the physical exercise program performed by us, which will be mentioned during the review, was a regular and moderate intensity aerobic exercise (defined as training), consisting of 12 weeks (1 h/day, 3 times/week) of swimming program, voluntary, for both the male obese diabetic rats (ZDF *fa/fa*) and the male control lean animals (ZDF *+/+*), between 8 and 20 weeks of age [32-34]. In brief, the protocol used was: the animals, maintained under controlled temperature (22°C), humidity (60%) and lighting (12 h of light) conditions, given a rodent maintenance chow (A-04 Panlab, Barcelona, Spain) adjusted to their respective weights (100 mg/g of weight) and distilled water ad libitum, perform their exercise in a cylindrical tank, 120 cm in diameter and 80 cm in height, containing water with a controlled temperature (30 -32°C); the animals were placed in the tank every day at the same hour (09.00 -10.00 h) under the supervision of the same person; the swimming period was initially for 15 min/d and was gradually increased such that the rats were able to perform exercise for 60 min/d, which was achieved in 1 wk; after 1 wk of this training period, the rats were made to swim for 1 h, three times a week; at the end of each exercise session, the animals were dried and kept in a warm environment; the sedentary rats were kept in the container where the swimming sessions were held for a period of 60 min to ensure that these control rats underwent the same amount of stress as the test animals that performed exercise. The animals that practiced exercise were sacrificed 48 h after the end of the last training session to minimize the acute effects of the exercise. The night before sacrifice, food was removed from the animal cages.

### Physical activity, obesity and body fat distribution

Our studies showed that exercised diabetic rats presented, when sacrificed 48 h after the last bout training session, a trend to increase body weight, which might be due to an increase in muscle mass [34]. Despite the lack of measurement of the animal body fat amount, a reduction in total visceral or subcutaneous fat in exercised animals cannot be excluded. Similar effect was observed by other studies in humans, confirming that after the training there was an increase in muscle mass with decrease in fat mass [35,36].

In the same work, Teixeira de Lemos et al. [34] showed that the weight of some organs or tissues (heart, liver, kidneys and muscle) were heavier in the exercised diabetic rats when compared with the sedentary animals, thus confirming that training leads to important morphological and physiological adaptations to maintain body homeostasis, as previously suggested by others [37,38]. In addition, the results suggest that the maintenance in time of training is an important factor for the appearance of those adaptations.

The study conducted by Tuomilehto et al. (2001) provided evidence that T2DM, in both women and man at high cardiovascular risk, can be prevented by lifestyles modifications, with a decrease of overall incidence of diabetes of 58% [39]. Regarding physical exercise practice, which has included components designed to improve both cardiorespiratory fitness and muscle strength, the results showed that more than 4 h/week of exercise was associated with a significant reduction in risk of diabetes even without weight loss [39]. Some of the key beneficial effects of an exercise program include visceral obesity reduction and muscle mass increase. Randomized control trials conducted in individuals with normal body mass index (BMI), as well as in patients with abdominal obesity and T2DM, demonstrated that physical exercise regularly practiced contributes to diminish total, visceral and subcutaneous fat, even without weight loss, together with improvement of glycaemia and with increase of FFA oxidation and, thus, to an amelioration of the diabetes [40-42].

### Physical exercise and glycaemia and insulinaemia control

The first aim of T2DM treatment is hyperglycaemia control, as a way of reducing chronic diabetic complications, namely of cardiovascular nature. The American Diabetes Association (ADA) recommends a value of HbA1c above 7%. Our group demonstrated, using the training protocol above described in ZDF (*fa/fa*) rats, that hyperglycaemia was prevented by exercise, together with a significantly lower value of HbA1c (-6,6%), when compared to sedentary counterpart, reinforcing the idea of a effect maintained over time [33,34]. This results were corroborated by Kyraly et al. (2008) in ZDF rats submitted to forced swim training (1 h/day; 5 days/week during 13 weeks) [43]. Additionally, in our study the hiperinsulinaemia was partially, but significantly, corrected in the trained rats, which was accompanied by reduction of insulin resistance, given by the lower HOMA (homeostasis model assessment), and index of insulin resistance. Thu, we hypothesize that swimming training was able to improve peripheral insulin resistance, although the less action on hepatic resistance, suggesting that hyperinsulinaemia could be a reflex of insulin resistance in the liver, not improved by exercise [33,34].

Concerning studies in humans, in a meta-analysis which reviewed the studies concerning exercise intervention of at least 8 weeks in type 2 diabetic individuals, regular aerobic exercise showed a statistically and clinically significant effect on HbA1c, suggesting that this non-pharmacological intervention improve glycaemic control, while having little effect on body weight [44]. Similar results were encountered in another meta-analysis on the effect of exercise practice, which included 14 studies (12 with aerobic exercise and 2 with resistance exercise) [45], demonstrating that the effect of exercise on HbA1c (the major marker of glycemic control), is a well established finding.

The amelioration on glucose metabolism by exercise training may occur primarily through three distinct mechanisms: i) stimulation of glucose transport to muscle; ii) increased in insulin action on cells of the organs involved in the exercise; iii) positive regulation of signaling pathway stimulated by insulin as a result of regular exercise.

Exercise has been indicated as an "insulin-like" activity because of the increase of muscle's capacity to capture circulating glucose, due to decreased intramuscular fat reserves [40]. Christ-Roberts et al. (2004) found that exercise training significantly increased expression of GLUT4 glucose transporter in overweight nondiabetic and diabetic subjects, by 38% and 22%, respectively [46,47]. Akt protein expression, which was decreased by about 29% in the diabetic subjects before training, when compared to the nondiabetics, increased significantly in both groups [46]. Furthermore, it was also observed that in skeletal muscle exercise training affects the transcriptional regulation of the gene of the IRS-1 and the post-transcriptional regulation of the PI3-kinase expression [48,49]. The increased capacity of the muscle to oxidize fat in response to aerobic exercise is also a major mechanism by which exercise training improves insulin sensitivity in the muscle [50]. Taken together, the above mentioned actions of exercise (training) on skeletal muscle contribute to regulate blood glucose levels.

### Exercise and dyslipidaemia

Chronic exercise (training) has favorable effects on lipid profile [34,51], being nowadays viewed as one of the best non-pharmacological strategies for the prevention or attenuation of diabetic dyslipidaemia. Our group demonstrated that aerobic exercise training improved dyslipidaemia in ZDF rats, namely by reducing the total-cholesterol (T-Chol) and triglycerides (TGs) [34]. Among other benefits, exercise stimulates lipolytic activity (with decreased plasma TG), promotes the use of FFA as an energy source and increases HDL concentration. Furthermore, favorable changes in the quantity and composition of LDL particles were also shown, as well as on the quality of HDL [52,53]. The primary mediator mechanism of these changes seems to be the beneficial influence of regular exercise on the activity of peripheral enzymes, such as lipoprotein lipase (LPL), lecithin-cholesterol acyltransferase (LCAT) and hepatic lipase (HL) [51]. In addition to the regulation of the mechanism of hepatic lipid transformation, moderate physical exercise increases the oxidative capacity of several tissues, including the skeletal muscle, which is under low oxidative capacity in situations of insulin resistance. Physical exercise increases the number of capillaries and oxidative fibers in muscle, increasing lipolysis, which allows free flow of fatty acid to the tissue, reducing its concentration in plasma, which is an indicator of its uptake and oxidation by tissues [54].

It seems clear now that regular exercise training is able to improve lipid metabolism. But is this evident in human studies? Type 2 diabetes populations have been shown to improve fasting blood lipid profile following long-term exercise interventions, with or without dietary restriction [55,56]. Furthermore, exercise practice in Type 2 diabetes patients showed improved glycemic control, body composition, blood pressure, muscle strength, and workload capacity, together with attenuated progressive increase in exogenous insulin requirements [57]. In accordance with earlier reports, the randomized trial conducted by Sigal et al. (2007) showed that, despite an unaltered body weight, combined endurance and resistance type of exercise training is able to induce regional changes in fat and lean muscle mass in obese T2DM patients [58]. Furthermore, Lira et al. (2007) also reported that low and moderate exercise intensities (training) appear to promote clear benefits on lipid profile [59].

The exercise is also able to activate an alternative pathway: the AMPK [60]. This enzyme acts on the liver, muscle and adipocytes by increasing fatty acid oxidation, decreasing cholesterol synthesis, lipogenesis and lipolysis, and even modulating insulin secretion on pancreatic islets [61]. Apart from the effect that AMPK appears to have on lipid oxidation, it also plays an important role in decreasing the glucose levels, being able to stimulate GLUT-4 increment [62].

Considering the data above mentioned, it seems obvious that the regular practice of an exercise program has a positive effect on the dyslipidaemic profile displayed by patients with T2DM whic could not be neglected.

### Physical exercise and blood pressure

It is widely accepted that the exercise practiced on a regular basis has an antihypertensive effect in humans [63,64]. Indeed, regular exercise (training) is able to reduce heart rate, improving the sensitivity of aortic baroreceptors, which contributes to a more efficient regulation of blood pressure [65]. The beneficial effects on hypertension (blood pressure lowering, either systolic or diastolic) due to decreased activity of both the sympathetic nervous system and the renin-angiotensin system was also documented. Other mechanisms responsible for the antihypertensive effect of training include the decrease in peripheral arterial resistance caused by vasodilatation [66]. Besides improving glycaemic control, a meta-analysis showed that structured exercise intervention studies in non-insulin-dependent Type 2 diabetes patients reduce systolic blood pressure of about -4.16 mmHg [67]. Such reduction in mean blood pressure are clinically relevant and similar to the effects produced by combined therapy of an angiotensin-converting enzyme (ACE) inhibitor and an thiazide diuretic [68].

Also in animals, as shown by our studies using the ZDF rats as model of type 2 diabetes, training (swimming) has promoted a decrease in systolic and mean blood pressure and in heart rate, together with a diminishment of differential pressure [33,34], suggesting an improvement of vascular arterial compliance, with reduction in cardiac work and a left ventricular hypertrophy amelioration. The increased arterial stiffness appears to be one of the factors that best combine cardiovascular risk and atherosclerosis. Differential pressure has been indicated as an indirect measure of arterial stiffness and a better predictor of coronary risk. By preventing the increasing of differential pressure regular exercise training positively influence the cardiovascular diabetic complications, such as diabetic ischemic heart disease, which is often asymptomatic.

## Exercise (training), oxidative stress and T2DM

### Exercise and oxidative stress - pathophysiological aspects

Exercise is associated with increased formation of free radicals, mainly due to increased O2 consumption by active tissues. Several studies have shown that the amount of free radicals in biological tissues is increased after acute and/or chronic exercise, which coincides with the presence of tissue damage [69]. Most of the O2 consumed is used in the mitochondria for oxidative phosphorylation, where it is reduced to water. However, a small but significant fraction of O2 consumed may leave the electron transport chain to produce ROS; it is estimated that approximately 2-5% of oxygen used by mitochondria are converted into free radicals [70].

Chronic exercise of moderate intensity (training) positively alters the oxidative homeostasis of cells and tissues, by decreasing the basal levels of oxidative damage and increasing resistance to oxidative stress [71]. In fact, regular exercise causes adaptations in the antioxidant capacity, protecting cells against the harmful effects of oxidative stress, thus preventing cellular damage [72,73]. Adaptation to oxidative stress in trained individuals is clearly evidenced by a decrease in DNA damage, by sustained levels of protein oxidation and by an increment of resistance against chronic administration of hydrogen peroxide [74]. Training is also able to alter the metabolism of purines, reducing the availability of substrate for xanthine oxidase (XO) in the trained muscle and plasma content of hypoxanthine and uric acid.

### Exercise and oxidative stress in T2DM

Oxidative stress has often been implicated in the pathogenesis of micro and macrovascular diseases observed in diabetic individuals. Some data support a role of regular exercise in reducing lipid peroxidation. Indeed, if regular exercise can show a protective effect against oxidative stress in individuals with diabetes mellitus, their use, as a non-pharmacological therapeutic measure for T2DM, become even more attractive

The cardiovascular adaptations observed by practicing regular physical exercise (training) include, as above mentioned, not only lowering blood pressure, but also aggregation and adhesiveness of platelets and increment of cardiac blood flow [75,76]. These adaptations may be mediated, at least in part, by a hyper-regulation of basal nitric oxide (NO) production. Consistent with this idea are the reports of increased NO production in subjects who practiced chronic exercise, coincident with decrease in blood pressure and platelet activation [77]. The augment of NO production observed during acute exercise is able to induce protective adaptations by interaction with various transcription factors and, thereby, influence gene expression of antioxidant enzymes [78].

Although antioxidant properties have been attributed to acid uric, high level of uric acid is strongly associated, and in many cases predicts, development of hypertension, visceral obesity, insulin resistance, dyslipidaemia, T2DM, kidney disease, and cardiovascular events [79,80]. Several studies suggest that, under certain concentrations, uric acid might have antioxidant activity, preventing lipid peroxidation; nevertheless, its association with chronic disease highlights the uric acid oxidant-antioxidant paradox [81]. Ideally, exercise training should be able to reduce pro-inflammatory levels of uric acid to anti-oxidant and protective levels. Considering the negative consequences associated with oxidative stress, our group demonstrates in diabetic ZDF animals submitted to a swimming training protocol an increased antioxidant enzyme activity, with concomitant decline in oxidative aggression [34]. This effect of training might suggests a beneficial regulation of XO activity, which might be viewed as a possible therapeutic strategy for treatment diabetes-associated diseases [80].

**Figure 1:**
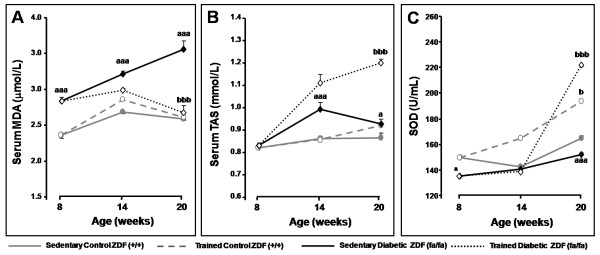
**Evolution of serum MDA (A), TAS (B) and blood SOD (C) levels between T0 and Tf in control (+/+) and diabetic (*fa/fa*) Zucker diabetic fatty rats: control sedentary (black circles), control exercised (white circles), diabetic sedentary (black diamonds) and diabetic exercised (white diamonds) **. Data are means ± sem of eight separate values (rats) per group. Statistical significance: ^aaa ^P < 0.05: sedentary diabetic vs sedentary control; ^b^P < 0.05 and ^bbb^P < 0.001: exercised control or diabetic vs sedentary control or diabetic, respectively. MDA, malondialdehyde; SOD, superoxide dismutase; TAS, total antioxidant status. Adapted from [32].

In our animal studies, using the ZDF rat, the exercise-induced oxidative injury decrease was accompanied by an augmentation in serum total antioxidant status (TAS) and in superoxide dismutase (SOD) activity (Figure 1), reinforcing the antioxidant action of training. Furthermore, the decline observed in 3-nitrotyrosine (3-NT) serum levels of trained diabetic rats suggests a decrease in peroxynitrite contents, corroborating the work of Fukai et al. (2000), which demonstrated that training promotes the increase of endothelial nitric oxide syntase (eNOS) gene expression and its phosphorylation, thus protecting endothelial cells [82].

## Exercise (training), inflammation and T2DM

### Exercise and inflammation - pathophysiological aspects

According to Kasapis et al. (2005), a single session of exercise triggers an increase in pro-inflammatory cytokines release, associated with leukocytosis and increased plasma concentration of C-reactive protein (CRP) [83]. This pro-inflammatory response to acute exercise is accompanied by a sudden increase in oxidative stress and, followed by adaptive mechanisms against inflammation [84]. Moreover, longitudinal studies showed that regular training induces a reduction in CRP levels, suggesting an inflammatory action, viewed in several conditions, such as T2DM, insulin resistance and other cardiovascular/cardiometabolic diseases [84]. Regular exercise is associated with a decrease of CRP, IL-6 and TNF-α levels and, simultaneously, with increase of anti-inflammatory substances, such as IL-4 and IL-10 [84], reinforcing the anti-inflammatory nature of exercise [85,86].

Cytokines are released not only from mononuclear cells but also from muscle cells. Starkie et al. (2003) showed that physical exercise directly inhibits endotoxin-induced TNF-α production in humans, most likely through IL-6 release from exercising muscle [87]. Typically, IL-6 is the first cytokine present in circulation after exercise practice, followed by an increase in IL-1ra and IL-10 [88]. The ubiquitous role of IL-6 and the hypothesis of an exercise-induced anti-inflammatory IL-6 release was recently reviewed [89,90]. Therefore, IL-6, a multifactorial cytokine, regulates cellular and humoral responses and plays a pivotal role in inflammation, being associated with several pathological conditions, including type 2 diabetes, emerging as an independent early predictor for T2DM and as a marker of low-grade inflammation [89,90]. However, what is even more interesting concerning IL-6, as Fisman and Tenenbaum (2010) recently commented, is the putative beneficial effects played as an anti-inflammatory factor, which is particularly evident in insulin sensitivity during exercise [89]. Therefore, a marked increase in circulating levels of IL-6 after exercise without muscle damage has been a remarkably consistent finding. The magnitude by which plasma IL-6 increases is related to exercise duration, intensity of effort, muscle mass involved in the mechanical work and endurance capacity [91]. The release by muscle of a humoral factor into the circulation after exercise improves insulin sensitivity, most probably through AMPK [89]. IL-6 has been indicated as the strongest candidate for the humoral factor released after exercise, working in a hormone-like fashion, in which it is released by the muscle, now viewed as an endocrine organ, for influencing other organs [89]. Although this hypothesis deserve further clarification, the role of IL-6 as both the "good" and the "bad", depending on the circumstances, as commented by Fisman and Tenenbaum (2010), opens new windows on the way interleukins act, and in particular concerning the effects of exercise in insulin resistance and diabetes. In this anti-inflammatory environment, IL-6 inhibits TNF-α production, which was confirmed by studies in animals [92]. Furthermore, exercise also suppresses secretion of TNF-α by pathways independent of IL-6, as shown by the results obtained with knockout mice for IL-6 submitted to exercise [93]. The anti-inflammatory nature of regular exercise (training) has been associated to a reduced cardiovascular disease, particularly due the training-evoked increased expression of antioxidant and anti-inflammatory mediators in the vascular wall, which could directly inhibit atherosclerosis development [94].

The information now available concerning the effects of physical exercise on adiponectin levels is scarce and divergent [95]. There were several studies that showed that chronic exercise (programs of 6 weeks to 6 months) did not induced changes in adiponectin levels [96]. Kriketos et al. (2004) also reported, after 2-3 sessions of moderate exercise, a remarkable increase in adiponectin levels (260%), that remaining elevated for 10 weeks, without body weight modifications [97]. The systematic review performed by Simpson and Singh (2008), considering literature searches databases conducted from ten years and including 8 randomized controlled trials, concluded that exercise of varying prescription increase serum adiponectin in 38% of de trials, demonstrating small-to-moderate effect sizes [95]. Nevertheless, the same study showed inconsistent data in the literature for increasing adiponectin levels after short-term exposure to robust aerobic or resistance training of moderate-to-high intensities, reinforcing the need of more studies reporting consistent findings concerning a clear relationship between changes in adiponectin contents and exercise mode, intensity and frequency [95].

### Exercise and inflammation in T2DM

The above data highlighted the idea that beneficial effect of exercise seem to be related to its ability to decrease inflammatory cytokines levels and/or increase anti-inflammatory ones, which might be also true for pathological conditions, such as type 2 diabetes.

**Figure 2:**
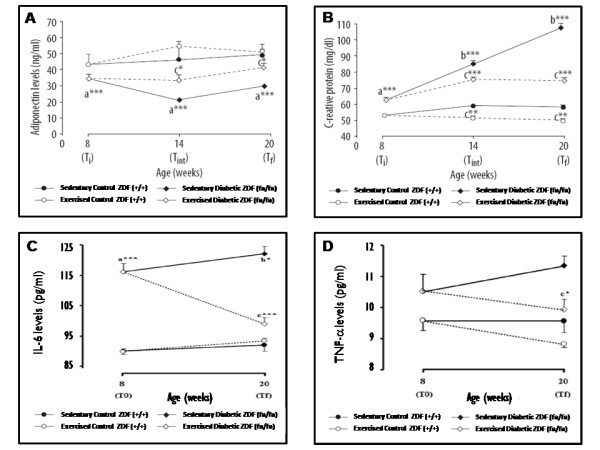
**Evolution of serum adiponectin (A), C-reactive protein (B), IL-6 (C) and TNF-α (D) levels between T0 and Tf in control (+/+) and diabetic (*fa/fa*) Zucker diabetic fatty rats: control sedentary (black circles), control exercised (white circles), diabetic sedentary (black diamonds) and diabetic exercised (white diamonds)**. Data are means ± sem of eight separate values (rats) per group. Statistical significance: ^a ^*fa/fa *versus +/+; ^b ^Tf versus T0; ^c ^exercised rats versus sedentary rats; *P < 0.05, **P < 0.01 and ***P < 0.001. IL-6, interleukin 6; T0, initial time; Tf, final time; TNF-α, tumor necrosis factor-α. Adapted from [[33] and [[34]].

The results from the studies of Teixeira de Lemos et al., above mentioned [33,34], clearly demonstrated the anti-inflammatory capacity of swimming exercise training in the ZDF rat, a model of obese T2DM. Actually, training was able to prevent the increase of pro-inflammatory cytokines and CRP observed in the diabetic rats. Those findings were in the line of those of Martin-Cordero et al. (2009), which found that obese Zucker rats, a model of metabolic syndrome, presents impairment of pro-inflammatory cytokines (TNF-α, IL-6, IL-1beta and interferon gamma: IFN-γ) release by macrophages, an effect that was improved by habitual physical activity [98,99]. Furthermore, Teixeira de Lemos et al. also found an increment of serum adiponectin in trained obese diabetic ZDF (fa/fa) rats to levels nearby those found in the control lean rats (Figure 2). Adiponectin anti-inflammatory actions has been associated with improvement of cardiometabolic profile, which might be due, at least in part, by regulatory actions on other factors, including on TNF-α, IL-6 and CRP levels [100], which was also demonstrated in our study using the ZDF rat submitted to swimming regular exercise training [33,34] (Figure 2). Considering that adiponectin measure was performed 48 hours after the last training session, the results may suggest an extension of the anti-inflammatory effect obtained by a single bout of exercise.

Pancreatic islets from type 2 diabetic patients present amyloid deposits, fibrosis and increased cell death, which are associated with the inflammatory response [101]. T2DM is also characterized by hyperglycemia, dyslipidaemia, increased circulating inflammatory factors and cellular stress, which are critical in precipitating islet inflammation in vivo. Chronic exposure of β cell to these mediators induces excessive production of ROS and activation of caspases, which inhibit insulin secretion and promote apoptosis of pancreatic β cells [102]. The impact of islet-derived inflammatory factors and islet inflammation on β-cell function and mass may be both beneficial and/or deleterious. Depending on their roles in regulating pancreatic β-cell function, some cytokines are protective while others can be detrimental. Actually, chronic exposure of islets to IL-1β, IFN-***γ***, TNF-α and resistin inhibits insulin secretion and induces β cells apoptosis. Other cytokines, such as adiponectin and visfatin, exert protective effects on pancreatic β cell function. In addition to circulating cytokines, islets also produce a variety of cytokines in response to physiologic and pathologic stimuli, and these locally produced cytokines play important roles in regulation of pancreatic β-cell function as well [103]. To maintain the normal pancreatic β-cell function, the deleterious and protective cytokines need to be balanced. The abnormal control of cytokine profile in islets and in plasma is associated with pancreatic β-cell dysfunction and type 2 diabetes [103]. All those emerging evidences reinforce the paradigm that islet inflammation is involved in the regulation of β-cell function and survival in T2DM.

**Figure 3:**
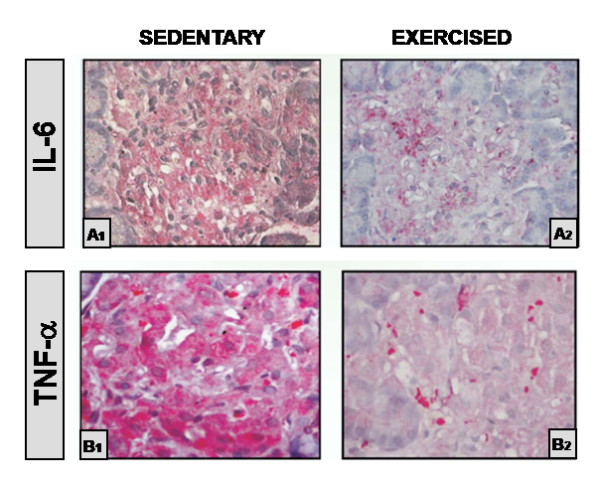
**Interleukin-6 (A) and TNF-α (B) immunostaining of islets of Langerhans (original magnification 400×) in ZDF rats**. (A1 and B1) - Staining of the islet of a 20-wk-old sedentary diabetic (fa/fa) rat showing high immunostaining (+++); (A2 and B2) - Staining of the islet of a 20-wk-old exercised diabetic (*fa/fa*) rat with a significant reduction in the expression of interleukin-6 (A) and TNF-α (B) immunoreactivity (+).IL-6, interleukin 6; TNF-α, tumour necrosis factor α. Adapted from [34]).

Few studies have previously reported the putative beneficial effects of regular exercise practice (training) on pancreas, *per se*. Studies in Otsuka Long Evans Tokushima Fatty (OLETF), Goto-Kakizaki (GK), Zucker fatty (ZF) and ZDF rats have shown improvements in whole-body insulin sensitivity and preservation of β-cell mass with exercise training [104,105]. Insulin sensitivity improvements by exercise may confer an indirect beneficial effect on β-cells by decreasing insulin demand and minimizing β-cell exhaustion, at the same time minimizing hyperglycemia mediated loss in β-cell function [106], but a direct effect on pancreatic function could not be excluded. Although almost all the studies have demonstrated β-cell mass preservation with exercise training, none of them focus on inflammation. The recognition that islet inflammation is a key factor in TD2M pathogenesis has highlighted the concern regarding the protection of pancreatic islets and endocrine function. Thus, restoring the normal cytokine profile in endocrine pancreas and plasma may hold great promise for more efficient β-cell dysfunction treatment and T2DM management. Teixeira de Lemos et al. [34] demonstrated, using the above mentioned animal model of obese T2DM, the ZDF rat, that exercise training was able to prevent accumulation of pro-inflammatory cytokines (IL-6 and TNF-α) on endocrine pancreas (Figure 3). A decrease in pancreas immunostaining of both cytokines was observed, suggesting a protective effect of regular physical exercise against local inflammation.

## Final remarks and conclusions

The recognition that a tight glycaemic control significantly reduces the microvascular and macrovascular complications in T2DM indicates hyperglycaemia as the main goal of treatment. Indeed, the reduction in HbA1c values was shown to have a positive impact on cardiovascular complications associated with diabetes. Epidemiological data from the UKPDS (United Kingdom Prospective Diabetes Study) suggest that the reduction of blood glucose decreases the risk of cardiovascular disease, which was supported by recent meta-analyses that concluded by a beneficial impact of glycaemic control in reducing events of non-fatal myocardial infarction and events of coronary heart disease, despite no significant effect on all-cause mortality [107,108]. Thus, nevertheless the key role of hyperglycaemia lowering in T2DM management and prevention of its serious complications, the correction of other associated risk factors, such as dyslipidaemia, hypertension, hypercoagulability, obesity and insulin resistance, is also crucial for better efficacy of treatment.

The current therapeutic arsenal for treatment of T2D is mainly based on:

i) - reduce hepatic glucose production (metformin);

ii) - stimulate insulin secretion (sulfonylureas, glinides);

iii) - delay the intestinal glucose absorption (alpha-glucosidase inhibitors);

iv) - increase sensitivity of muscle, fat and liver to insulin (Thiazolidinediones);

v) - suppress glucagon secretion and delay gastric emptying [Glucagon-like peptide-1 (GLP-1) agonists];

vi) - extend GLP-1 activity after meals in order to reverse the failure of pancreatic beta cells [Dipeptidyl peptidase-4 (DPP-4) Inhibitors];

vii) - stimulate peripheral glucose uptake and decrease hepatic glucose production (insulin).

In addition, for the correction of other risk factors encountered in T2DM patients, other drugs are also requested:

i) - lipid lowering drugs (statins, ezetimibe, fibrates or combinations);

ii) - antihypertensive drugs [ACE inhibitors, angiotensin II receptor antagonists (ARAs), beta blockers, diuretics, calcium entry blockers];

iii) - antiplatelet drugs [acetylsalicylic acid (ASA), clopidogel, triflusal or associations].

A recent proposal to condense into a single drug more active principles (polypill), as a tool for primary and secondary prevention of cardiovascular disease and T2DM evolution, is, in theory, apparently very attractive. However, apart from the putative side effects and the so large range of possible drug-to-drug interactions, an antidiabetic polypill will need to be adapted to one or more stages of diabetic dysmetabolism, which is a progressive disease. The large size of formulations, as well as the loss of flexibility of the therapy, which is essential for controlling metabolic changes and to handle variations in blood pressure, should be also be carefully considered.

**Figure 4:**
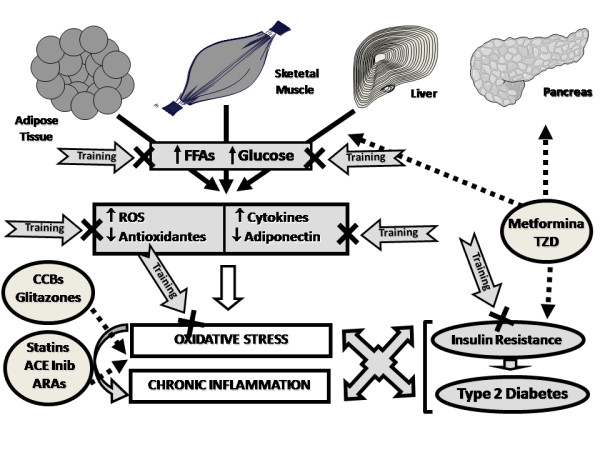
**Diagram illustrative of the pleiotropic effect of regular physical exercise (training) as an antidiabetic "Polypill"**. ACE, angiotensin-converting enzyme; ARAs, angiotensin II receptor antagonists; CCBs, calcium channel blockers; FFA, free fatty acids; ROS, reactive oxygen species; TZD, Thiazolidinediones.

Throughout this document, which reviewed the beneficial effects of regular exercise on the correction of risk factors for T2DM, the similarities between the effects of chronic exercise and a putative antidiabetic polypill were highlighted, with the additional advantage that exercise, when practiced regularly and under moderate intensity (training), do not causes relevant side effects and presents a greater metabolic effectiveness if compared with an antidiabetic polypill (Figure 4).

The studies developed by our group [32-34], using an animal model of obese T2DM, clearly demonstrated that the practice of a regular and moderate intensity swimming protocol (training), although unable to fully reverse pancreas lesions, has prevented and/or delayed the worsening of diabetic dysmetabolism. The obtained results highlighted the pleiotropic effect of exercise training, viewed by several properties, including:

i) - improvement of arterial vascular compliance and blood pressure;

ii) - correction of dysglycaemia and dyslipidaemia;

iii) - increment of antioxidant defenses, thus promoting a reduction of oxidative aggression;

iv) - decrease of pro-inflammatory profile and increased anti-inflammatory markers;

v) - reduced pancreatic dysfunction in Langerhans islets, responsible for the cell failure and appearance of relative insulin deficiency with insulin resistance, a feature of advanced stages of T2DM.

The cardiometabolic protective role of exercise training in T2DM becomes more clear when considering the pleiotropic actions encountered by our group, which were corroborated by other studies in animal models, as well as in humans, as above commented in detail, together with other important action that undoubtedly contribute to prevent or attenuate diabetes evolution and its complications, which includes:

- accentuation of the reduced myocardial β-adrenergic responsiveness in diabetic rats, mainly due to the reduction in β2-adrenoceptors expression, which might have protective action [109];

- decreases in resting systolic blood pressure and 24-hour proteinuria in obese diabetic patients with chronic kidney disease (CKD), which is in favour of reduced cardiovascular complications in these patients [110];

- reduction in plasma endothelin 1 (ET-1) and NO content, together with beneficial effects on anthropometric measurements and plasma oxidant stress markers, suggesting an improvement of endothelial dysfunction in patients with IGT [111];

- improvement of TNF-α and IL-6 release impairment by non-infiltrated peritoneal macrophages in a rat model of obese metabolic syndrome [98,99].

Considering the data now reviewed, exercise prescription might be recommended as adjuvant of drug therapy for treatment/attenuation of T2DM and its serious complications, which is in line with the recommendations of American diabetes Association (ADA) and European Association for the Study of Diabetes (EASD) algorithm for the management of type 2 diabetes, further strengthened by a possible reduction in the dose of anti-diabetic drugs, as well as of other drugs used to correct/attenuate the associated cardiometabolic risk factors. This data is even more relevant when recognizing that the epidemic of obesity and insulin resistance is already focused on children and adolescents. However, we must recognize that further research is needed, namely in humans, in order to establish the preferred type, duration and intensity of training that should be practiced in order to maximize the benefits of exercise for different subgroups of T2DM patients.

## List of Abbreviations

3-NT: 3-nitrotyrosine; ACE: angiotensin-converting enzyme; ADA: American Diabetes Association; AGE: advanced glycation endproducts; AMPK: 5' adenosine monophosphate-activated protein kinase; ARAs: angiotensin II receptor antagonists; ASA: acetylsalicylic acid; ASP: acylation-stimulating protein; BMI: body mass index; CCBs: calcium channel blockers; CKD: chronic kidney disease; CRP: C -reactive protein; DNA: deoxyribonucleic acid; DPP-4: dipeptidyl peptidase-4; EASD: European Association for the Study of Diabetes; eNOS: endothelial nitric oxide syntase; ET-1: endothelin 1; FFA: free fatty acids; GK: Goto-Kakizaki; GLP-1: glucagon-like peptide-1; GLUT4: glucose transporter type 4; HbA1c: glycosylated haemoglobin; HDL: high-density lipoprotein; HL: hepatic lipase; HOMA: homeostasis model assessment; IDF: international Diabetes Federation; IFN-γ: interferon gamma; IGT: impaired glucose tolerance; IL: interleukin; IR: insulin resistance; IRS-1: insulin receptor substrate-1; JNK/SAPK: kinases JunNH2-terminal/kinases of proteins activated by stress; LCAT: lecithin-cholesterol acyltransferase; LPL: lipoprotein lipase; MDA: malondialdehyde; NADPH: nicotinamide adenine dinucleotide phosphate; NF-kB: nuclear factor-kB; NO: nitric oxide; OLETF: Otsuka Long Evans Tokushima Fatty; ox-LDL: oxidized low density lipoproteins; p38MAPK: mitogen-activated protein kinase p38; PAI-1: plasminogen activator inhibitor 1; PI3K: phosphatidylinositol 3-kinase; PKC: protein kinase C; PPAR-α: peroxisome proliferators activated receptor alpha; RAGE: related to receptor for AGE; ROS: reactive oxygen species; SOD: superoxide dismutase; T2DM: type 2 diabetes mellitus; TAS: total antioxidant status; T-Chol: total-cholesterol; TGs: triglycerides; TNF-α: tumour necrosis factor α; TZD: Thiazolidinediones; UKPDS: United Kingdom Prospective Diabetes Study; VEGF: vascular endothelial growth factor; XO: xanthine oxidase; ZDF: Zucker Diabetic Fatty; ZF: Zucker fatty

## Competing interests

The authors declare that they have no competing interests.

## Authors' contributions

ETL, SN and FR drafted the manuscript. FT and FR critically reviewed the manuscript. All authors read and approved the final manuscript.
